# Visual Fixation and Continuous Head Rotations Have Minimal Effect on Set-Point Adaptation to Magnetic Vestibular Stimulation

**DOI:** 10.3389/fneur.2018.01197

**Published:** 2019-01-22

**Authors:** Bryan K. Ward, David S. Zee, Dale C. Roberts, Michael C. Schubert, Nicolas Pérez-Fernández, Jorge Otero-Millan

**Affiliations:** ^1^Department of Otolaryngology-Head and Neck Surgery, The Johns Hopkins University, Baltimore, MD, United States; ^2^Department of Neurology, The Johns Hopkins University, Baltimore, MD, United States; ^3^Department of Neuroscience, The Johns Hopkins University, Baltimore, MD, United States; ^4^Department of Ophthalmology, The Johns Hopkins University, Baltimore, MD, United States; ^5^Department of Physical Medicine and Rehabilitation, The Johns Hopkins University, Baltimore, MD, United States; ^6^Department of Otolaryngology, Clinica Universidad de Navarra, Madrid, Spain

**Keywords:** magnetic vestibular stimulation, MRI, vision, fixation, labyrinth

## Abstract

**Background:** Strong static magnetic fields such as those in an MRI machine can induce sensations of self-motion and nystagmus. The proposed mechanism is a Lorentz force resulting from the interaction between strong static magnetic fields and ionic currents in the inner ear endolymph that causes displacement of the semicircular canal cupulae. Nystagmus persists throughout an individual's exposure to the magnetic field, though its slow-phase velocity partially declines due to adaptation. After leaving the magnetic field an after effect occurs in which the nystagmus and sensations of rotation reverse direction, reflecting the adaptation that occurred while inside the MRI. However, the effects of visual fixation and of head shaking on this early type of vestibular adaptation are unknown.

**Methods:** Three-dimensional infrared video-oculography was performed in six individuals just before, during (5, 20, or 60 min) and after (4, 15, or 20 min) lying supine inside a 7T MRI scanner. Trials began by entering the magnetic field in darkness followed 60 s later, either by light with visual fixation and head still, or by continuous yaw head rotations (2 Hz) in either darkness or light with visual fixation. Subjects were always placed in darkness 10 or 30 s before exiting the bore. In control conditions subjects remained in the dark with the head still for the entire duration.

**Results:** In darkness with head still all subjects developed horizontal nystagmus inside the magnetic field, with slow-phase velocity partially decreasing over time. An after effect followed on exiting the magnet, with nystagmus in the opposite direction. Nystagmus was suppressed during visual fixation; however, after resuming darkness just before exiting the magnet, nystagmus returned with velocity close to the control condition and with a comparable after effect. Similar after effects occurred with continuous yaw head rotations while in the scanner whether in darkness or light.

**Conclusions:** Visual fixation and sustained head shaking either in the dark or with fixation inside a strong static magnetic field have minimal impact on the short-term mechanisms that attempt to null unwanted spontaneous nystagmus when the head is still, so called VOR set-point adaptation. This contrasts with the critical influence of vision and slippage of images on the retina on the dynamic (gain and direction) components of VOR adaptation.

## Introduction

People working around strong magnetic resonance imaging (MRI) machines have reported transient sensations of rotation (1–3). A key to understanding the physiology underlying this effect is observed when visual fixation is removed. A horizontal nystagmus was first reported in a 1.5T magnetic field when fixation was removed (4) and a persistent, higher-intensity horizontal (and torsional) nystagmus is seen in humans when in darkness in magnetic fields of higher strengths [3-7T (5, 6)]. Although the sensation of rotation fades away within several minutes, some nystagmus persists inside the MRI up to the longest time tested thus far of 90 min (7, 8). This nystagmus induced by the magnetic field of the MRI can be suppressed with visual fixation upon a target. A key feature of a nystagmus that originates from labyrinthine imbalance is suppression of the nystagmus during visual fixation.

The proposed mechanism for the MRI-induced nystagmus and vertigo is a Lorentz force, generated by interactions between the flow of ions through inner ear endolymph into utricle hair cells and the strong static magnetic field of the MRI machine (6). This Lorentz force is proportional to the strength of the magnetic field, the net current flowing into hair cells and the height over which the current travels. As long as the subject remains in the MRI, the Lorentz force displaces the cupula of the lateral and superior semicircular canals (6), causing vertigo and nystagmus and creating an effect similar to constant acceleration of the head (8).

Many agonist/antagonist systems exist within the body that allow quick responses to environmental changes; and these systems operate around a balanced level of tonic activity—the set-point—providing, a stable platform from which they generate a response. Set-point adaptation is the process by which the brain modifies a system's tonic activity in response to a sustained change in the environment. As an agonist/antagonist system the vestibulo-ocular reflex (VOR) operates around a stable set-point. By inducing a constant displacement of the semicircular canal cupulae and observing the dynamics of the VOR, we can study set-point adaptation (9). Over time in the MRI the nystagmus slowly but only partially decays, implying incomplete adaptation. Upon exiting the magnetic field an after effect appears in which the nystagmus and sense of rotation reverse direction. The presence of the after effect reflects adaptation that has occurred in the MRI. We previously showed that the time course of adaptation to magnetic vestibular stimulation (MVS) can be described by a set of adaptation operators of increasing time constants working in parallel (8). It is unknown, however, what error signals are responsible for driving these different adaptation processes.

In the case of adaptation of the dynamic components of VOR [e.g., (10)] retinal slip (motion of the images upon the retina) is an important error signal that informs when the eye has moved an incorrect amount to compensate for the head movement. In the case of set-point adaptation during MVS with visual fixation, retinal slip could also indicate a wrong set-point when there is a nystagmus. However, because the nystagmus is greatly suppressed retinal slip might not be an explicit error signal for set-point adaptation. Instead the mechanisms that suppress the nystagmus, e.g., the motor commands that enable steady fixation, may interact with the adaptation processes.

Multiple systems can use visual information and contribute to the suppression or cancellation of an undesired nystagmus due to an imbalanced vestibular system. The smooth pursuit system generates eye movements that maintain the image of a moving object of interest on the center of the fovea. The optokinetic system monitors full-field visual motion to generate eye movements that work in concert with the VOR to maintain a stable retinal image during head movements. Both systems could cooperate to suppress the nystagmus although there might also be a specific fixation system that keeps the eyes still (11).

Here we sought to determine whether visual fixation influences the early phases of VOR set-point adaptation in order to infer where along the neural pathway the components of set-point adaptation are occurring. Because of spontaneous nystagmus, the obvious error signals to drive set-point adaptation are retinal slip, and the brain's attempt to eliminate it. Hence, we first asked if visual fixation would hasten the rate of adaptation.

## Methods

Eye movements were tracked using video oculography on six individuals before, during and after exposure to a 7T MRI scanner (Philips Research, Hamburg, Germany). There were four men, two women; ages 33 to 71 years. In all experiments subjects were supine on the MRI table and entered the MRI bore in the head-first position. The magnetic field vector *B* of the MRI in this study was directed from the subject's head toward the feet when entering the MRI head-first. Eye movements were recorded with infrared illumination using the RealEyes xDVR system (Micromedical Technologies Inc.) and custom software to measure binocular eye position in three dimensions (12). As part of this system, video is recorded at 100 Hz using separate cameras for each eye (Firefly MV, Point Grey Research Inc., Richmond, BC, Canada). During dark conditions, vision was occluded with a double layer of black felt to ensure complete darkness. During visual fixation conditions, after entering the magnet in darkness subjects removed the black felt, the MRI bore lights were turned on, and the subject was instructed to fix their gaze at the intersection of a vertical and horizontal black line (21.6 × 27.9 cm) on the inside surface of the magnet bore (distance of approximately 30 cm). To assess whether there were differences with the type of visual stimulus used, additional experiments were performed with a rich visual stimulus of yellow dots (each 2 cm diameter) randomly positioned on a black background that filled most of the visual field (55.9 × 71.1 cm). Following the fixation portion of the trial, the lights were turned off, and subjects replaced the black felt over their eyes.

**Figure 1:**
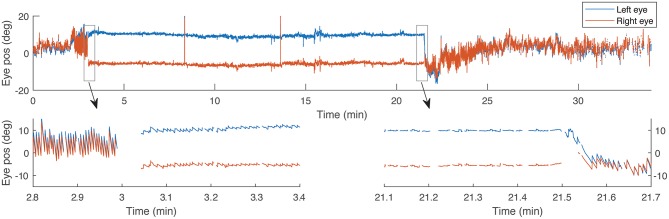
An example recording of horizontal eye position during a 20-min trial with visual fixation. Bottom panels show details of 30-s time periods around the times when the light turns on and off, respectively. Gaps in the data correspond with eye blinks.

Trials began with the subject supine on the MRI table with their head near the bore in a neutral position, and eye movements were recorded for 2 min outside the MRI. Subjects entered the MRI at a fixed-speed of 10.8 cm/s over the 2 m travelled to the center of the bore. Eye movements were recorded for 5, 20, or 60 min and then subjects exited the MRI and recording continued for an additional 4, 15, or 20 min. For each subject the trial was then repeated entering the magnetic field in darkness, followed by light with visual fixation, beginning 60 s after entering the magnetic field. Subjects were placed in darkness again 10 s before exiting the bore (30 s in the 20- and 60-min trials). All subjects performed visual fixation with intersecting lines. Three subjects also underwent trials with the full-field stimulus. To assess whether fixation had an impact on later components of adaptation, three subjects underwent five trials lasting 20 min in the MRI and one subject underwent a trial lasting 60 min. Figure 1 shows an example of eye movement recording during a 20-min visual fixation trial.

Additionally, a subset of subjects (*n* = 2) performed the above series of experiments, except that instead of the second series of trials using a visual fixation stimulus, yaw head rotations (~2 Hz) in the dark while inside the MRI were performed, starting 60 s after entering the magnetic field and stopping 10 s prior to exiting the MRI bore. Head rotations were kept at a constant rate using the periodic noise of the MRI machine (~2 Hz) as a metronome. These trials of yaw head rotations were repeated in the light with subjects fixing on a full-field stimulus.

The velocity of the slow-phase component of nystagmus (SPV) was calculated in 1 s windows. First, we identified outliers in the eye movement recordings due to blinks and other eye tracking artifacts as portions of the data with large and fast changes in estimated pupil size (more than 10% around a 5 s average), too high velocities (1,000 deg/s), or accelerations (50,000 deg/s^2^). Then, we detected and removed quick-phases to calculate the SPV. In a first pass we detected quick-phases as portions of the eye velocity with speeds above 100 deg/s. Then, we removed those portions of data with an additional 30 ms before and calculated a smooth version of the velocity using a median filter (4 s window). Next, we subtracted this smooth version of the velocity from the original eye velocity and ran a second pass of quick-phase detection, this time with a more sensitive 10 deg/s threshold. After removing the newly detected quick-phases (and 30 ms before and after) from the raw velocity we applied another median filter (1 s window) on the remaining data to obtain the slow-phase velocity. Finally, we averaged the slow-phase velocity of the two eyes and applied an additional median filter to reduce the effect of potential noise present in only one eye. Eye movements when the head was rotating were removed and not analyzed.

The amplitude of the after effects were compared for all subjects between trials with visual fixation and those in darkness by calculating the area under the curve of the after effect within a 3 min window after exiting the magnet (7 min window for 20 min trials). Statistics were performed using a paired *t*-test and a *p*-value of <0.05 was considered significant. For graphs of averages across subjects, SPV traces were first normalized by dividing the SPV by the peak SPV during the corresponding dark condition for that subject. Experiments were approved by the Johns Hopkins institutional review board and written informed consent was obtained from all participants.

## Results

**Figure 2:**
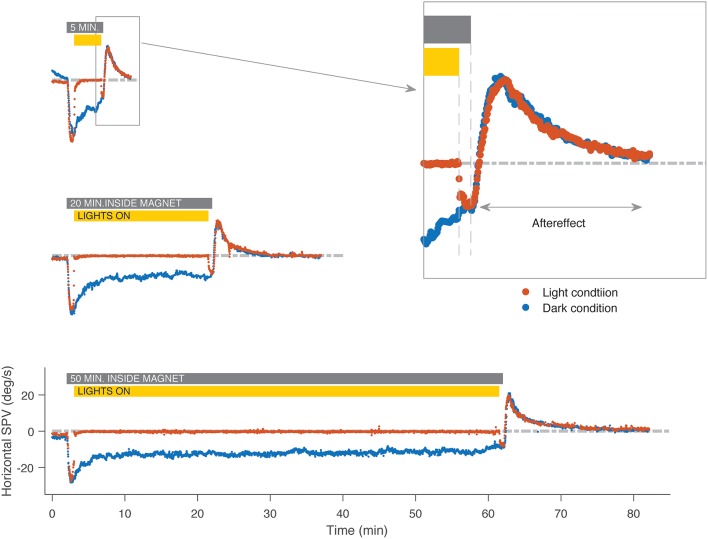
An example subject's horizontal slow-phase nystagmus eye velocity in the MRI. The blue traces represent trials in darkness without visual fixation and the red represent trials where the subject enters the MRI in darkness, lights are turned on for visual fixation, and then turned off before exiting from the MRI. In darkness, a pattern of first fast then slow adaptation occurs, followed by an after effect in the opposite direction. Aside from the period of visual fixation, the slow-phase velocity is similar, regardless of whether lights were on or off, or of the duration in the MRI. Gray bar indicates the time the subject was inside the magnet for both trials in darkness and with visual fixation, and yellow bar indicates duration of exposure to visual fixation in the light for trials with fixation only. SPV, slow-phase eye velocity.

**Figure 3:**
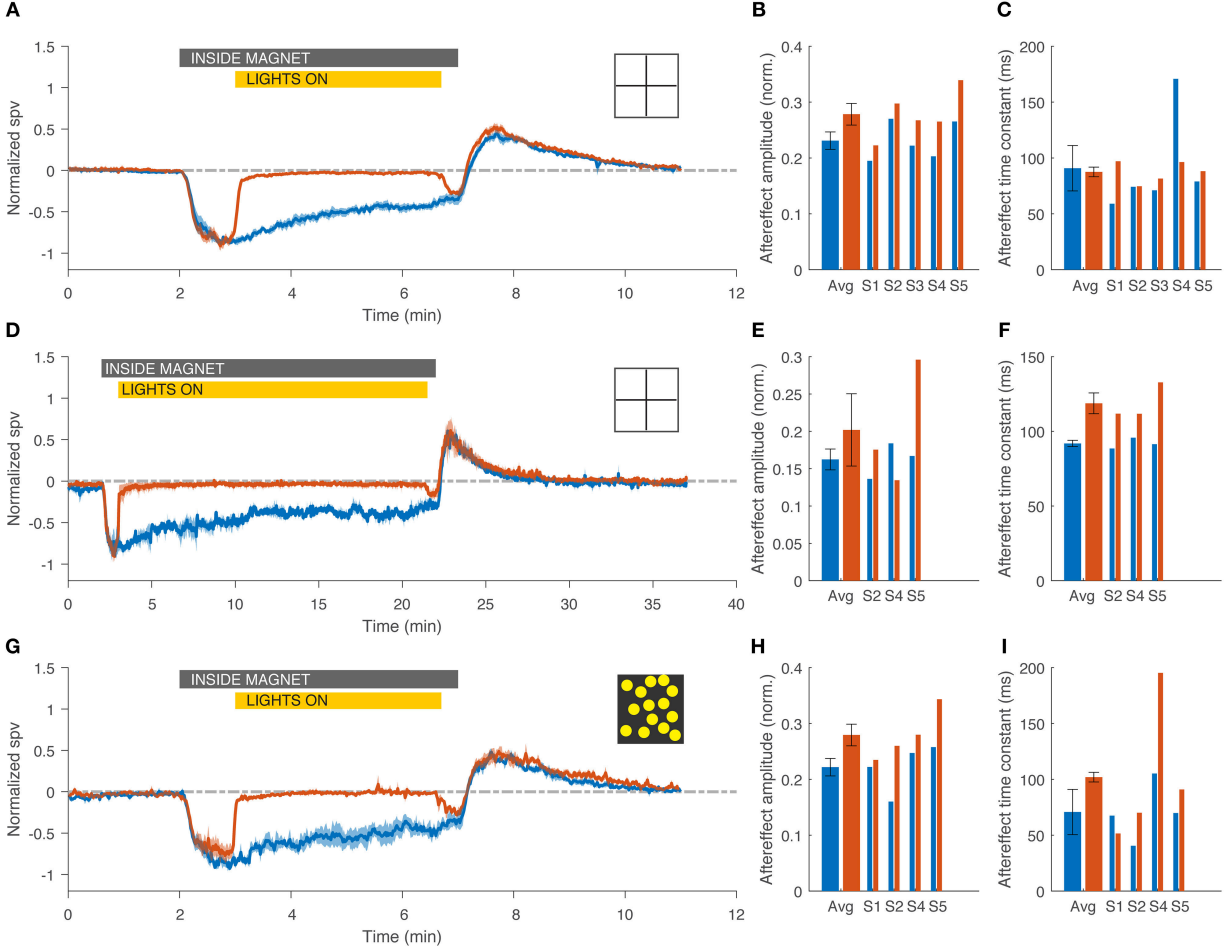
Visual fixation had little impact on the adaptation to the magnetic vestibular stimulus, regardless of the duration of fixation (top and middle rows, 5 and 20 min) or the visual fixation stimulus (top and bottom rows, 5 min, simple cross and rich full field stimulus). **(A,D,G)** Average normalized slow-phase velocity (SPV) across subjects for fixation (red) and darkness (blue) conditions. Note the different time scale on the abscissa for the graph in **D**. **(B,E,H)** Amplitude of the after effect, calculated for each subject as the area under the curve during the after-effect period (3 min after exiting the magnet for the 5 min trials and 7 min for the 20 min trials). **(C,F,I)** Time constant of the decay of the after effect, calculated with an exponential fit starting at the maximum SPV during the after effect. Gray bar indicates the time the subject was inside the magnet for both trials in darkness and with visual fixation, and yellow bar indicates duration of exposure to visual fixation in the light for trials with fixation only. Bar graphs show average and individual results. Errors bars and shaded area represent standard error of the mean (SEM).

As prior studies, all subjects developed a horizontal, and torsional nystagmus when entering the magnetic field in the head-first position in darkness. The nystagmus slowly, but incompletely faded in the MRI, and reversed direction upon exiting the magnetic field (Figure 2, blue traces). With visual fixation in the MRI, all subjects could suppress the spontaneous nystagmus that had been observed in darkness (Figure 2, red traces) down to a slow-phase velocity of −0.6 ± 0.3 deg/s. When subjects removed fixation while still within the magnetic field, a horizontal and torsional nystagmus resumed, and upon exiting the magnetic field there was an after effect. The amplitude of the after effect, reflecting adaptation inside the MRI was slightly increased with fixation in the short duration trials (*N* = 5, *p* = 0.007 Figures 3A,B), but not in the longer duration trials (*N* = 3, *p* = 0.5, Figures 3D,E) or the trials using a full-field visual stimulus (*N* = 4, *p* = 0.06, Figures 3G,H). The time constant of the decay of the after effect was not different between trials with and without visual fixation in the short duration trials (*N* = 5, *p* = 0.8, Figure 3C), the long duration trials (*N* = 3, *p* = 0.07, Figure 3F), nor in the trials using a full-field visual stimulus (*N* = 4, *p* = 0.3, Figure 3I).

**Figure 4:**
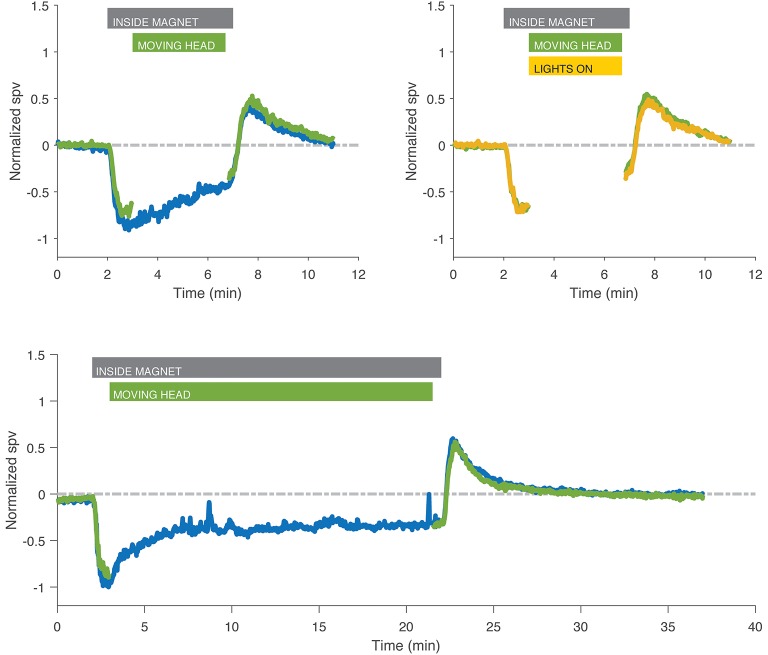
Recordings with head rotations at ~2 Hz in the MRI while in darkness (green) or with fixation (yellow) compared to control experiment in darkness without head movement (blue) in two different subjects. Eye movements during the head shaking have been removed for clarity. Head rotations, either in darkness or with fixation, had no effect on the after effect compared to lying still in darkness, nor was there any effect that might have been superposed from a head-shaking induced nystagmus. Gray bar indicates the time the subject was inside the magnet for both trials in darkness and with visual fixation, green bar indicates the time the subject was moving the head, and yellow bar indicates duration of exposure to visual fixation in the light for trials with fixation only.

For subjects that performed the sustained head rotations inside the MRI, the velocity of nystagmus at the time of stopping head movements and the after effect after exiting the magnetic field were the same as from trials in which the subjects lay in the field in darkness and also the same as trials in which subjects performed sustained head rotations with the lights on and a full-field visual stimulus (Figure 4).

**Figure 5:**
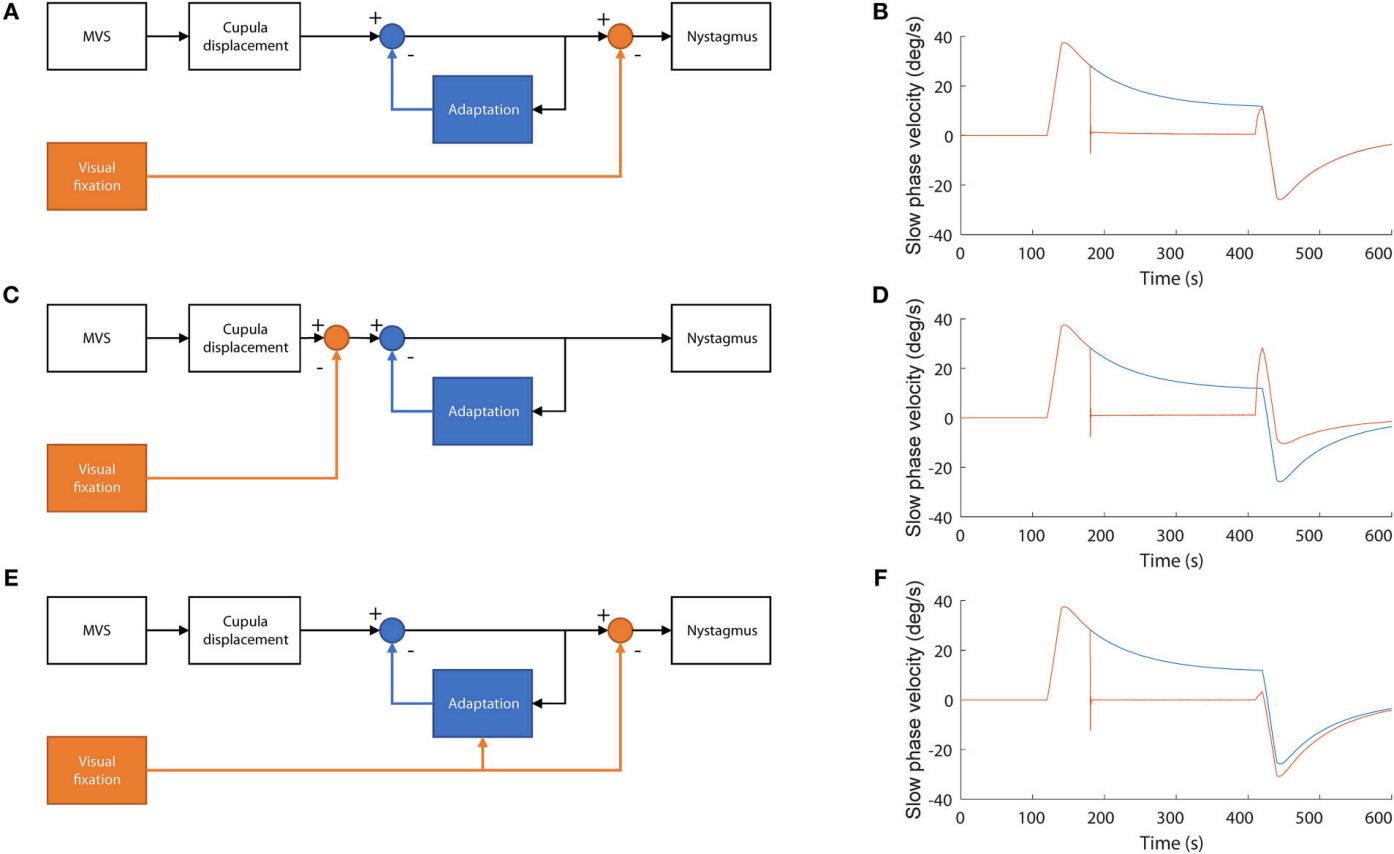
Simulations of alternative models of the interaction between adaptation and the cancellation of nystagmus by visual fixation. **(A,B)** Visual information is used to cancel nystagmus at a stage of processing later than adaptation. In this model adaptation is not affected by the presence of visual fixation while in the MRI. This simulation accords best with our data **(C,D)** Visual information is used to cancel the nystagmus at a stage before adaptation occurs. In this case, no adaptation occurs during fixation and thus the after effect is smaller, only due to the initial first minute of adaptation in darkness, **(E,F)** Visual information is used to cancel nystagmus after adaptation occurs but also to enhance adaptation. In this case, adaptation is stronger, and the after effect is larger.

There are multiple ways in which visual information can affect adaptation, depending on when the suppression of the nystagmus occurs relative to adaptation and on whether visual information itself can interact with the adaptation processes. To test which of the scenarios better corresponded with our data, we simulated the system under different configurations using a control-systems approach (see Supplementary Table 1 and Supplementary Figure 1). We started with a model of MVS set-point adaptation (8) and added a mechanism for visual suppression of nystagmus based on a simple smooth pursuit system that cancels the retinal slip of the fixation target (Figure 6) from Robinson et al. (13). Then, we compared the simulated slow-phase velocity for conditions equivalent to our MVS recordings under different model configurations. First (Figures 5A,B), adaptation occurs at an earlier stage than cancellation of nystagmus by the VOR. Second (Figures 5C,D), adaptation occurs after the cancellation of nystagmus by the VOR. Third (Figures 5E,F), adaptation occurs earlier than the cancellation of the nystagmus by the VOR but it is also enhanced when visual information is available. These simulations suggest our data are consistent with visual fixation suppressing the nystagmus at a later stage than adaptation and with little or no interaction between visual suppression and the adaptation operators.

## Discussion

We found that adaptation to a magnetic field-induced, static vestibular imbalance and the adaptation after effect when the subject came out of the magnetic field, were nearly superimposable regardless of whether the eyes were fixing on a target or the subject was in darkness while in the MRI machine. This occurred regardless of whether a relatively simple target or a rich visual stimulus occupying the entire visual field was used. Likewise, head rotations while in the MRI machine did not influence the adaptive process. A magnetic vestibular stimulus, generated by the interactions of a strong static magnetic field and the natural ionic currents of the inner ear, is thought to induce a constant displacement of the semicircular canal cupulae (5, 6, 14). This stimulus generates eye movement responses similar to those expected for a constant acceleration stimulus (8). A nystagmus of peripheral origin is suppressed by visual fixation and becomes apparent when visual fixation is eliminated. The finding here of consistent suppression of the nystagmus with visual fixation supports a peripheral origin for magnetic vestibular stimulation. Perhaps surprisingly, the presence of vision, while suppressing the nystagmus response, had little impact on the rate or amplitude of adaptation, or on the after effect, suggesting that the early process that monitors asymmetric vestibular input is occurring largely independent of vision. Jareonsettasin et al. found that the two early components of adaptation to a static vestibular imbalance have time constants of 1 to 2 min and 10 to 20 min while the late component has a time constant of more than 1 h (8). Our combined results for 5, 20, and 60 min suggest that the presence of vision does not impact either of the first two components of adaptation.

### MVS as a Technique to Explore Set-Point Adaptation

In dynamic vestibular adaptation of the gain of the VOR, motion of an image on the retina is thought to be the error signal used by the cerebellum to recalibrate the VOR to ensure a clear image during head movements. After a lesion to the peripheral vestibular system, this restoration of the gain of the VOR toward normal tends to gradually occur over time (15). Similarly, for static, set-point adaptation of the VOR, there must exist a mechanism that monitors the spontaneous neural discharge occurring at the vestibular nuclei in order to rebalance activity between the two sides. For example, in the case of a patient who undergoes a labyrinthectomy or vestibular neurectomy, a spontaneous nystagmus will develop that will adapt slowly over days to weeks (16, 17). Normal subjects show adaptation that occurs within minutes during a sustained, constant-velocity or constant acceleration rotation of the head (18, 19). The nystagmus generated by magnetic vestibular stimulation also adapts slowly but incompletely in the longest tested trial of 90 min (8). Presumably the nystagmus would eventually disappear over a longer time period. The after effect observed after trials of magnetic vestibular stimulation, is proportional to the duration of adaptation that has occurred when in the MRI, and is absent for short duration exposures (6). Magnetic vestibular stimulation therefore is a useful, and in many ways ideal model to explore the mechanisms of the relatively early phases of adaptation to a static vestibular in both normal human subjects and patients with vestibular lesions.

### The Role of Vision in VOR Set-Point Adaptation

By assessing the influence of vision on set-point adaptation to a vestibular imbalance, we can infer where along the adaptation pathway vision might have an effect. Despite vision and motion of images on the retina being critical signals for adaptation of the dynamic components of the VOR, our data show they have little if any have little if any effect upon the early components of VOR set-point adaptation. After acute unilateral labyrinthectomy in the monkey, an absence of vision does not prevent the eventual disappearance of the spontaneous nystagmus (20). Furthermore, occipital lobectomy in monkeys affects the dynamic component of vestibular adaptation, but not the static component (21), supporting not only that vision is unnecessary for adapting to a static vestibular imbalance, but also that the mechanism of set-point adaptation may be occurring earlier, in the brainstem or cerebellum. In the cat, however, Courjon et al. suggested that spontaneous nystagmus after a hemi-labyrinthectomy may fade more quickly if the cat is exposed to light; however, much of the nystagmus subsided even when the cats were kept in darkness (22).

### The Cerebellum in Set-Point Adaptation

The cerebellum may be involved in the process of static vestibular adaptation (23) and examples such as control of posture, pointing, and alignment of the eyes have suggested that the cerebellum facilitates set-point adaptation (24–26). Some insight can be gained from studies of what is called Bechterew's phenomenon, a spontaneous nystagmus that develops when a patient or animal loses labyrinthine function, first on one side, and then, after an interval, on the other side (27, 28). Without the presence of peripheral vestibular input, individuals develop a spontaneous nystagmus after injury to the second side that reflects the adaptation that had occurred between the injuries. The Bechterew's phenomenon can be found in decerebrate animals and in animals in which the cerebellum was removed, and is thought to reflect adaptation at the level of the vestibular nuclei (29).

### The Importance of the Vestibular Nucleus in Set-Point Adaptation

Additional animal studies also have suggested that set-point adaptation is occurring in the vestibular nucleus. After a vestibular lesion, numerous processes occur at the vestibular nucleus ranging from changes in gene expression, levels of neurotransmitters, inflammatory responses, and synaptic activity, among others (30). A loss in excitatory input from the lesioned vestibular afferents leads to a decrease in discharge at the level of the ipsilateral vestibular nucleus (31). A candidate location for the mechanism that rebalances activity is therefore at the level of the commissural system interconnecting the vestibular nuclei (20, 32). The commissural system contributes further inhibition to the ipsilateral vestibular nucleus, and as the resting discharge at the vestibular nucleus increases, behavioral compensation occurs (33). McCabe et al. performed a systematic series of lesion studies to determine the site responsible for rebalancing afferent activity of the medial vestibular nuclei, ultimately concluding that cerebellum, cerebrum, spinal cord, contralateral vestibular nuclei, and ipsilateral superior and lateral vestibular nuclei were unlikely to be the source, suggesting that the adaptation is happening early in the pathway, perhaps in the ipsilateral vestibular nucleus (34). Their findings support earlier work by Spiegel and Demetriades that the ipsilateral vestibular nucleus is critical for this rebalancing process (29). Duensing and Schaefer identified neurons in the vestibular nucleus that behaved differently from peripheral vestibular afferents, responding in the same way to head rotations to either side (35). Whether such neurons could be associated with set-point adaptation is unknown, but the characteristics of their responses are what would be needed to compare activity between the vestibular nuclei on both sides. Vestibular efferent neurons in frogs also respond in a similar manner, being excited in response to angular rotation to either side (36). Although the role of the vestibular efferents in monkeys and humans is unclear, there is some evidence in mice that they participate in compensation (37) and may help resolve spontaneous nystagmus in mice (38) and cats (39). Finally, there is likely some adaptation in the vestibular periphery which may also contribute to rebalancing (40, 41).

### Horizontal Head Rotations Have Minimal Impact on Set-Point Adaptation

In a few of our experiments subjects continuously rotated their head in the yaw plane at ~2 Hz both while in darkness and in light with visual fixation. These head rotations did not affect the adaptive response, supporting the idea that the mechanism underlying set-point adaptation can extract and compensate for a persistent static bias in the face of changing dynamic vestibular signals. We also wondered if our normal subjects would show an effect on adaptation of a post head-shaking induced nystagmus much as shown by patients with a pathologically-induced asymmetry in vestibular tone. For head-shaking to cause a nystagmus the velocity-storage system must also be functioning (16). Thus, the lack of an effect of head shaking on adaptation could be due to a change in the velocity-storage mechanism during adaptation. Another possibility is that our subjects did not develop sufficient additional asymmetries in vestibular tone for the post head-shaking effect to be seen in our data.

### Limitations and Caveats

The results of this study only apply to the early components of set-point adaptation in healthy adults with intact labyrinths. There may be different neural substrates for the different components of set-point adaptation, some of which might be more influenced by visual fixation. The stimulus in magnetic vestibular stimulation is bilateral, affecting both labyrinths at the same time. The response of the brain to a constant stimulus when there is only one labyrinth, for example, after a labyrinthectomy, may differ compared to the responses seen during MVS. Finally, patients with a chronic, pathological imbalance in vestibular input may also behave differently from the healthy adults used here. Nevertheless, our results overall were remarkably consistent and suggest that the immediate adaptation to a vestibular imbalance occurs at an early stage, and likely at the level of the vestibular nuclei.

Another consideration is that there are many vestibular nuclei neurons (e.g., vestibular only (VO) neurons) that have vestibulospinal projections and probably drive vestibulospinal reflexes (42). Eye-head (EH) cells or flocculus target neurons (FTN) in the vestibular nucleus respond to both eye and head movements (43). Perhaps an explanation for the lack of an effect of vision or of head rotations on the early adaptation seen here is the fundamental need of the organism for vestibulospinal balance in order to maintain upright posture, regardless of vision or nystagmus. Another potential confound in our data is that during the periods of fixation subjects had to look at a near target that required them to converge their eyes to a different position than when they were in the dark. The similar after effects we observed with and without fixation suggest that any difference in eye positions did not have an effect on adaptation.

## Conclusions

In sum, these data support a model in which the effect of visual fixation and of sustained head rotations on spontaneous nystagmus are introduced only after vestibular set-point adaptation has taken place. We have previously shown that set-point adaptation of VOR in response to an MRI occurs over multiple time courses of increasing duration that can be approximated with multiple time constants of increasing amplitude (8). The findings here suggest that vision or head rotations have little impact on early (seconds to minutes) vestibular set-point adaptation. Whether or not vision influences the later (hours to days) components of set-point adaptation is unknown. Furthermore, these different adaptive processes may occur in different anatomic locations or by different mechanisms. Future knowledge on the substrates of adaptation may lead to ways to alter the time courses of adaptation favorably, allowing new treatments for human disease.

## Ethics Statement

Experiments were approved by the Johns Hopkins institutional review board and informed consent was obtained from all participants.

## Author Contributions

BW, DZ, MS, and JO-M designed the study. BW, DR, MS, and NP-F collected the data. JO-M and DZ developed the mathematical modeling. BW and JO-M wrote the initial drafts of the manuscript. All authors were involved in approval of the final version of the manuscript.

### Conflict of Interest Statement

The authors declare that the research was conducted in the absence of any commercial or financial relationships that could be construed as a potential conflict of interest.

## Supplementary Material

Supplementary Figure 1 To simulate different possible scenarios of the interaction between MVS and visual fixation we implemented a model in Simulink. The model only includes the horizontal canals and combines the left and right sides as a single control system that can encode positive and negative firing rates. The dynamics of the cupula include two force inputs: inertial force due to head acceleration (set to zero in this simulations) and the magnetic field induced Lorentz force. Then, there are two forces that counteract the movement of the cupula: viscous drag, proportional to velocity of the cupula and endolymph, and elastic force, proportional to the displacement of the cupula. The output of the cupula converted into firing rate units receives a positive feedback loop from the velocity storage system and two negative feedback loops from the adaptation operators. The output of the model is eye velocity which can be fed back to a simple system that attempts to cancel retinal slip (smooth pursuit or fixation system). We simulated three different configurations in this system: (1) if S1 switch is closed the eye velocity will be canceled by visual fixation after the adaptation occurs, (2) if S2 is closed the cancelation will occur before adaptation, and (3) if S3 is closed the visual system will enhance the vestibular adaptation by increasing its gain. The parameters of the simulations are included in Supplementary Table 1.

Supplementary Table 1 Simulation parameters.
