# Supplementary figures and images for: Visual Fixation and Continuous Head Rotations Have Minimal Effect on Set-Point Adaptation to Magnetic Vestibular Stimulation
Source: Front Neurol. 2019 Jan 22;9:1197. doi: 10.3389/fneur.2018.01197 (PMC6349782; doi:10.3389/fneur.2018.01197)

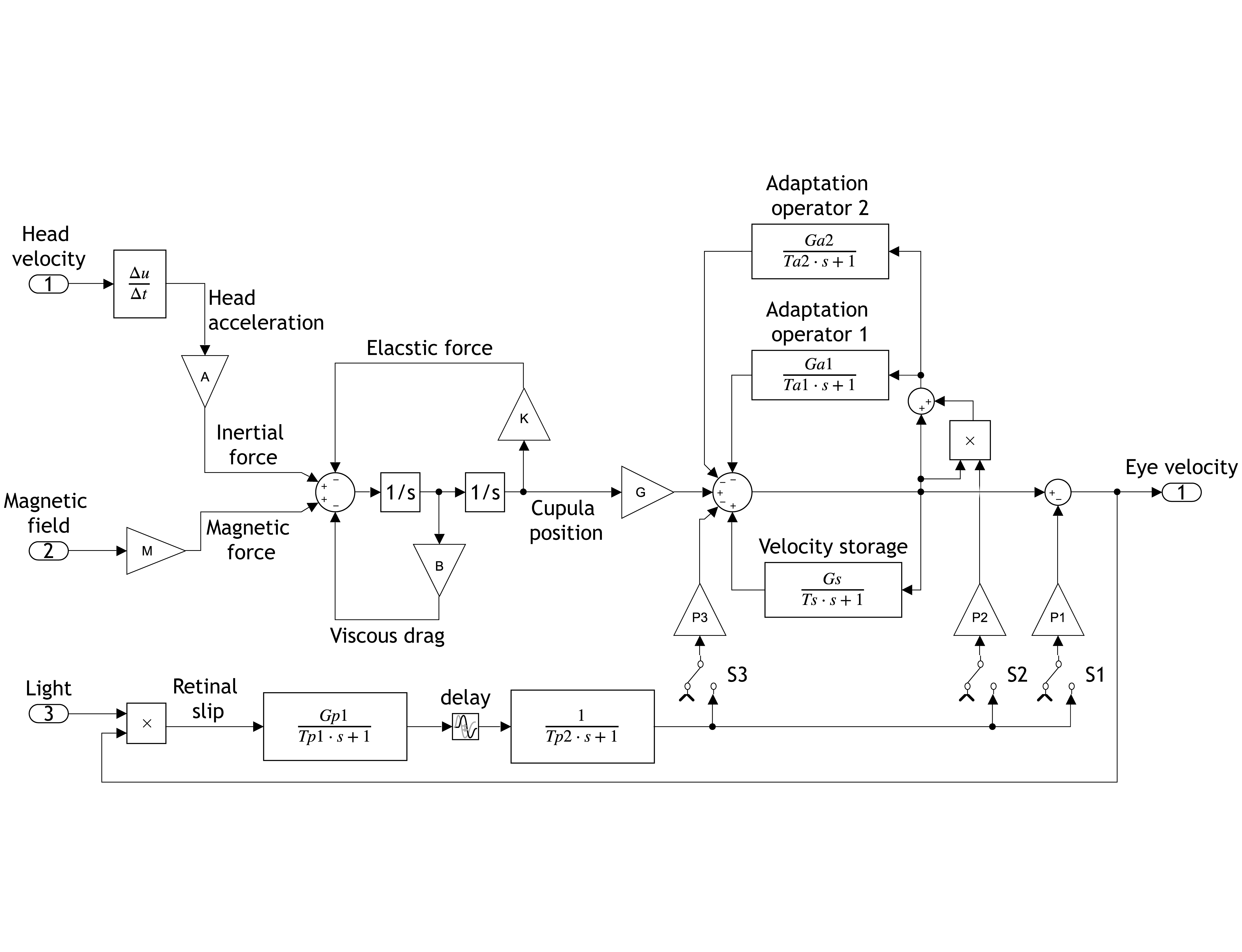

Supplement: Supplementary Figure 1 — To simulate different possible scenarios of the interaction between MVS and visual fixation we implemented a model in Simulink. The model only includes the horizontal canals and combines the left and right sides as a single control system that can encode positive and negative firing rates. The dynamics of the cupula include two force inputs: inertial force due to head acceleration (set to zero in this simulations) and the magnetic field induced Lorentz force. Then, there are two forces that counteract the movement of the cupula: viscous drag, proportional to velocity of the cupula and endolymph, and elastic force, proportional to the displacement of the cupula. The output of the cupula converted into firing rate units receives a positive feedback loop from the velocity storage system and two negative feedback loops from the adaptation operators. The output of the model is eye velocity which can be fed back to a simple system that attempts to cancel retinal slip (smooth pursuit or fixation system). We simulated three different configurations in this system: (1) if S1 switch is closed the eye velocity will be canceled by visual fixation after the adaptation occurs, (2) if S2 is closed the cancelation will occur before adaptation, and (3) if S3 is closed the visual system will enhance the vestibular adaptation by increasing its gain. The parameters of the simulations are included in Supplementary Table 1. [file Image_1.TIF]
