# Supplementary material for: Visual Fixation and Continuous Head Rotations Have Minimal Effect on Set-Point Adaptation to Magnetic Vestibular Stimulation
Source: Front Neurol. 2019 Jan 22;9:1197. doi: 10.3389/fneur.2018.01197 (PMC6349782; doi:10.3389/fneur.2018.01197)
Supplement: Supplementary Table 1 — Simulation parameters. [file Table_1.docx]

| **Parameter** | **Value** |
| --- | --- |
| A | 1 |
| M | 0.1 |
| B | 0.3 |
| K | 0.03 |
| G | 50 |
| Ga1 | 0.2 |
| Ta1 | 10 |
| Ga2 | 2 |
| Ta2 | 300 |
| Gs | 0.5 |
| Ts | 5.6 |
| Gp1 | 20 |
| Tp1 | 3.5 |
| Tp2 | 0.015 |
| delay | 0.13 |

**Table 1.** Simulation parameters.
